# Cross-country discrepancies on public understanding of stress concepts: evidence for stress-management psychoeducational programs

**DOI:** 10.1186/s12888-016-0886-6

**Published:** 2016-06-03

**Authors:** Juliana Nery Souza-Talarico, Nathalie Wan, Sheila Santos, Patrícia Paes Araujo Fialho, Eliane Corrêa Chaves, Paulo Caramelli, Estela Ferraz Bianchi, Aline Talita Santos, Sonia J Lupien

**Affiliations:** Department of Medical-Surgical Nursing, School of Nursing, University of São Paulo, Av. Dr Enéas de Carvalho Aguiar, 419, São Paulo, SP 05403 000 Brazil; Center for Studies on Human Stress, Mental Health Research Center Fernand-Seguin, Hospital Louis-H. Lafontaine, Université de Montreal, 7401, rue Hochelaga, Montréal, Québec H1N 3M5 Canada; Institute of Heart, Hospital das Clínicas, University of São Paulo, Av. Dr. Enéas de Carvalho Aguiar, São Paulo, SP 05403000 Brazil; Department of Internal Medicine, Behavioral and Cognitive Neurology Unit, Faculty of Medicine, Federal University of Minas Gerais, Av. Prof. Alfredo Balena, 190, Belo Horizonte, MG 30130-100 Brazil

**Keywords:** Psychological stress, Comprehension, Cross-cultural comparison, Social context

## Abstract

**Background:**

Negative effects of stress have pose one of the major threats to the health and economic well being of individuals independently of age and cultural background. Nevertheless, the term “stress” has been globally used unlinked from scientificevidence-based meaning. The discrepancies between scientific and public stress knowledge are focus of concern and little is know about it. This is relevant since misconceptions about stress may influence the effects of stress-management psychoeducational programs and the development of best practices for interventions. The study aimed to analyze stress knowledge among the Canadian and Brazilian general public and to determine the extent to which scientific and popular views of stress differ between those countries.

**Methods:**

We evaluated 1156 healthy participants between 18 and 88 years of age recruited from Canada (*n* = 502) and Brazil (*n* = 654). To assess stress knowledge, a questionnaire composed of questions regarding stress concepts (“stress is bad” versus “stress-free life is good”) and factors capable of triggering the stress response (“novelty, unpredictability, low sense of control and social evaluative threat versus “time pressure,work overload, conflict, unbalance and children”) was used.

**Results:**

Both Canadian and Brazilian participants showed misconceptions about stress and the factors capable of triggering a stress response. However, the rate of misconceptions was higher in Brazil than in Canada (*p* < 0.05).

**Conclusion:**

These findings suggest a lack of public understanding of stress science and its variance according to a country’s society. Psychoeducational programs and vulnerability of stress-related disorder are discussed.

**Electronic supplementary material:**

The online version of this article (doi:10.1186/s12888-016-0886-6) contains supplementary material, which is available to authorized users.

## Background

Over the last decades, the discussion about the “public understanding of science” has received special attention by scientistis and other professionals from the communication field, to increase public knowledge, access and use of scientific evidence. Regarding health science, the National Science Board of the United States revealed that only 40% of Americans know that “the father’s gene determines whether the baby is a boy or a girl”, and that half believe that “antibiotics kill viruses as well as bacteria” [1]. In addition, only 16.5% of Americans, on average, closely follow news about health, science and technology, while 48 percentage closely follow news on the weather [1].

Altogether, these evidence show that there is a lack of an integrated view of scientific knowledge in the general public.

In the field of stress science, the scenario is not different. For more than 70% of Americans, stress is caused by financial or workplace situations and 69% of parents claim their “stress has only a slight or no impact on their children” [2]. Popularly, stress is understood as time pressure. People usually perceived themselves stressed when the time available is not sufficient to perform a certain task or to achieve a goal. Additionally, people use to link stress to deleterious effects on health and seek to eliminate it from their lives leading to the understanding that stress is essencially harmful whatever the circunstances [3].

These findings show that there are misunderstandings and a lack of public awareness about the scientific definition of stress, the situations capable of eliciting a stress response, as well as its impact on the body and the brain of children, adults and older adults.

In a scientific perspective, stress is a body’s response that mobilizes energy necessary for fight-or-flight responses to real (e.g., extreme temperature, injury, or disease) and relative threat (dependent on individual interpretation) [3]. In the last case, the implied threat is interpreted according to the cognitive appraisal of situation demands and available resources [4, 5]. As such, stress is a highly personal experience because what is deemed stressful for one individual may not be by another [4, 5]. Regarding that, response-oriented stress theorists demonstrated that situations interpreted as novel, unpredictable, with no or low control or with a social evaluative component, whereby individual could be negatively evaluated by others or by self, are the psychological determinants capable of eliciting a stress response [6, 7]. Real and relative stressors activate sympathetic-adrenal-medullary (SAM) axis release of catecholamines and trigger the hypothalamic-pituitary-adrenal (HPA) axis to secrete cortisol, the main stress hormone in humans [8]. While acute stress response is adaptive in nature, chronic exposure to stress mediators from the prenatal period to aging impacts brain structures involved in cognition and mental health. Specific effects on brain, behavior and cognition emerge as a function of the stage of life and the duration of exposure to stress, and some of these effects depend on interaction between genes and exposure to environmental adversity [9]. For instance, repeated maternal stress during postnatal as well as child abuse, maternal separation allied to poor supportive care in the childhood is associated with altered cortisol levels that interfere in the HPA axis programming and developing. Accordingly adolescents who grew up in poor economic or adverse conditions have prolonged cortisol reactivity to acute stress that persists into adulthood potentiating the effects of further stress exposure. During adulthood and aging incubated effects of early adversity or maintenance of chronic stress associated with decline in the brain functioning make these group of individuals highly vulnerable to the negative effects of stress hormones [9].

There is a consensus that the mechanisms inherent to the stress response that aim to prepare and protect the organism and maintain stability (i.e., allostasis) can become deleterious for health and survival. This can occur whether called upon repeatedly in the form of chronic stress or in the presence of prolonged exposition to a stressor without adaptive behavior [10].

Chronic stress can lead to mental health problems such as depression, anxiety and burnout and stress *now* poses a major threat to the health and economic well being of individuals of all ages and all cultural backgrounds. In Canada, health problems as a result of increased stress at the workplace are steadily on the rise and other industrialized countries. Nearly 500,000 Canadians are absent from work each week due to stress-related health problems, and stress as a reason for absence from work has increased 316% since 1995 [11]. Overall estimates of the annual financial burden of stress-related mental health problems in Canada vary between $8 and 10 billion per year in absenteeism and $36 billion per year in presenteeism [12]. Similarly, 70 % of the economically active Brazilian population is stressed and 30 % of them present burnout. Workers who hold higher positions spend approximately 65 h per week at work. Nearly 75 to 90 % of the medical consultation is due to stress-related problems. Overall estimates of the stress-related problems are around 3.5 % of the Brazilian gross national product (around $42 billion per year) [13].

Based on these staggering costs of stress in the workplace, the World Health Organization now predicts that by the year 2020, depression will be the first cause of invalidity in the world followed by cardiovascular disease [14]. Depression and cardiovascular disease are two disorders that are tightly linked to chronic stress in humans.

Despite evidence regarding stress misconceptions [2], the public understanding of stress science has not been established among various populations across the world. This is important given that cultural beliefs, traditions, religious ideals, socioeconomic and sociopolitical characteristics could have a large impact on how mental health issues and stress are viewed across different cultures. Further, an understanding of how mental health and stress is viewed across cultures would be important for the development of appropriate educational programs and in the development of best practices for intervention with different cultures within any given country (immigration-based differences in stress conceptions). Sustaning this, changes in the conceptualization of stress has associated with improved cardiovascular, cognitive and affective responses to stress, decreasing the emotional impact of the stressful situation and exhibiting more adaptive responses [15–17].

In order to better understand public’s vision of stress, we investigated public knowledge of stress in Canada and Brazil. Canada is a developed nation while Brazil is an emerging economy country and member of BRICS (acronym that refers to the countries of Brazil, Russia, India, China and South Africa). Brazil has a large and fast-growing economy and significant influence on regional and global affairs yet it still presents substantial inequalities compared to developed nations [18]. Under different economic developments, Canada and Brazil present distinct socioeconomic, political and cultural characteristics capable of influencing the societies’ perceptions and knowledge on stress.

The objective of this study was to examine the understanding of stress among Canadian and Brazilian general public participants as well as to examine their knowledge of the characteristics that induce a stress response. Given that these two populations are exposed to different socioeconomic, politic and cultural environments, the scientific and popular view of stress in Canada and Brazil were compared.

## Methods

### Ethics statement

The study was approved by the Ethical Committee of the Research Centre of the Montreal Mental Health, University Institute, Montreal, Canada and by the Ethical Committee in Research of School of Nursing, University de São Paulo, São Paulo, Brazil. All participants signed an informed consent before the start of the study protocol.

### Participants

The present cross-country study was carried out in Canada at the Centre for Studies on Human Stress, University of Montreal and in Brazil at the Department of Medical-Surging Nursing, School of Nursing of the University of São Paulo and at the Internal Medicine at the Faculty of Medicine in the Federal University of Minas Gerais.

The sample composed of 1156 healthy participants between 18 and 88 years of age (M = 30.9, SD = 14.6) randomly chosen from the community in Montreal, Canada (*n* = 502, English speaking individuals) and São Paulo, Brazil (*n* = 654).

Participants from both groups had similar demographic characteristics except for age. Brazilian participants were younger than the Canadians. Demographic data are shown in Table 1.

**Table 1:** General sociodemographic, education and health indicators of Canada and Brazil

Characteristics	Canada	Brazil
Total population (in millions)	34,017	194,946
Population over 60 years (%)	20	10
Annual growth rate (%)	1.0	1.1
Life expectancy at birth (years)	81	73
Life expectancy at age 60 (years)	24	21
Literacy rate among adults aged ≥ 15 years (%)	---	90
Crude death rate (per 1000 inhabitant)	7.1	6.3
Gross National Income per capita (US$)	38,310	11,000
Population living below national poverty line (%)	9.4	26
*Health workforce*		
Physicians (per 10,000 inhabitant)	19.8	17.6
Nurses (per 10,000 inhabitant)	104.3	64.2
Hospital beds (per 10,000 inhabitant)	32	24
Mortality rate by cardiovascular disease and diabetes (ages 30 – 70 per 100,000 inhabitant)	82	248

*Source*: World Health Organization 2012 and World Development Indicators, World Bank 2012

Montreal is a metropolitan city in Canada, a developed country with sustained growth and economy, low birth rate, high life expectancy, high literacy level and trained workforce. São Paulo is the most populous city in Brazil with a population almost three-fold higher than Montreal. It has a rapidly emerging economy with a huge potential for additional growth, which nonetheless poses significant social inequalities including unemployment, unequal income, living conditions, and limited accessibility to health care and education [14]. In addition, Canadians and Brazilians are exposed to different patterns of historical social roles, values, norms and organization as well as language [18]. Sociodemographic, education and health indicators of Canada and Brazil are shown in Table 2.

**Table 2:** Demographic characteristics in Canada and Brazil

Variables	Canada (*n* =502) N (%) or Mean (±SD)	Brazil (*n* =654) N (%) or Mean (±SD)	p (value)*
Gender (female)	290.0 (57.8)	378.0 (58.3)	0.847^a^
Age (years)	37.3 ± 17.6	25.8 ± 8.9	<0.01^b^

^*^ Value of less than 0.05 indicates significance

^a^ Chi square test

^b^ *T*-Test

### Measures

Data collection was obtained using a general stress knowledge questionnaire that was created by the Centre for Studies on Human Stress, in Canada (Additional file 1). The 17-item self-applicable questionnaire took approximately 10 min to complete. These 17 questions were predominatly based on the stress concept (“stress is bad” *versus* “stress-free life is good”) and on the factors capable of triggering the stress response. Nine different factors were randomly listed in the questionnaire representing both the scientific psychological determinants (novelty, unpreditability, sense of control and threat to one’s ego) [6, 7] and the general public beliefs about stress factors (time pressure, work overload, unbalance between resources and demands, conflict and children).

Given that the original version of this instrument was created in English, the questionnaire was submitted to an adaptation process to the Brazilian population according the following stages: instrument translation from English to Portuguese, synthesis of the translated versions, back translation, analysis of the synthesized version by expert judges in the field of stress, and data collection. Within the validation process, a preliminary psychometric analysis was performed using Cronbach’s alpha coefficient (α) that yielded good reliability of the instrument for the total sample (Canadian and Brazilian; α = 0.724) and for the country group sample (Canadian x Brazilian; α for Canadian = 0.8; α for Brazilian = 0.714).

### Procedures

All participants were recruited and were evaluated in their own country according to the same protocol. The general stress knowledge questionnaire was given to both Canadian and Brazilian participants after the study’s objectives were explained.

### Statistical analysis

The quantitative variables were verified for assumption of normality. Group age differences were assessed using T-Tests. For dichotomic variables, the Chi square test was performed to compare frequencies. Furthermore, the independent variables country group (Canada *vs*. Brazil), age and gender (Male *vs*. Female) were input to a multiple linear regression model for each dependent variable (stress knowledge questionnaire itens - Yes *vs*. No) to ascertain stress misconceptions could be predicted by country group. All the dichotomous variables were “dimming coding” previously to the prediction analysis. The regression model was fitted using a backward method with unstandardized predicted values where a 0.10 entry value and 0.20 removal value were entered. The level of significance was set at 5 % (*p* < 0.05, 95 % confidence interval)

## Results

### Conceptions of stress

**Fig. 1:**
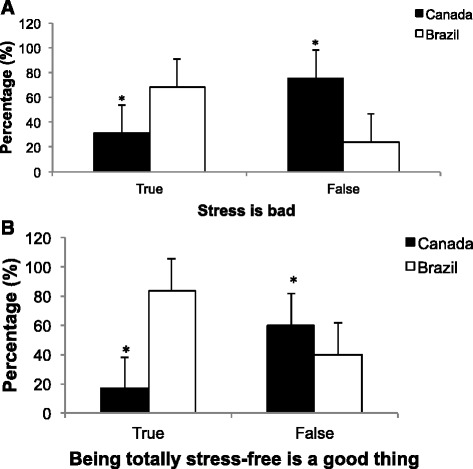
Percentage of participants in Canada and Brazil indicating stress to be bad (**a**) and being totally stress-free is a good thing (**b**). * indicates p-values less than 0.01 between groups. The bars represent standard errors

Participants were asked to indicate whether they viewed stress to be “bad” and whether they believed the phrase “being totally stress-free is a good thing” to be true or false. It is important to note that both these statements are false concepts according to the scientific stress response theory [3, 5–7]. Contrary to the Canadian group (*n* = 261; 54.5 %), more Brazilians reported that “stress is bad” (*n* = 581; 68.8 %; *p* < 0.001). Similarly, more Brazilians believed that “being totally stress-free is a good thing” (*n* = 365; 83.3 %), while the Canadian group reported an opposite view of this statement (*n* = 73; 14.7 %, *p* < 0.001). Figure 1.

Furthermore, multivariate analysis showed that country group influences the responses to both statements “stress is bad” (*p* < 0.001) and “being totally stress-free is good” (*p* < 0.001) even when age and gender were included in the model. Both country group and age explained 17 % of the variability in the “stress is bad” response, while approximately 22 % of the “stress-free is good” responses were due to country group and gender (Table 3).

**Table 3:** Multivariate regression results regarding stress conception

/Model	Stress is bad					Stress-free is good				
	β	p	R^2^	F	*p*	β	p	R^2^	F	*p*
*Model 1*			0.173	78.1	<0.001			0.217	104.6	<0.001
Country group	−**0.338**	<0.001				−**0.409**	<0.001			
Age	**0.148**	<0.001				0.041	0.167			
Gender	0.045	0.114				**0.201**	<0.001			
*Model 2*			0.171	115.8	<0.001			0.216	155.8	<0.001
Country group	**−0.343**	<0.001				**−0.425**	<0.001			
Age	**0.134**	<0.001				__^a^	__^a^			
Gender	__^a^	__^a^				**0.189**	<0.001			

^a^ Not included in the model 2 due to non significant effect in the model 1. Significant β values (*p* < 0.05) are in boldface

### Who is more “stressed”?

**Fig. 2:**
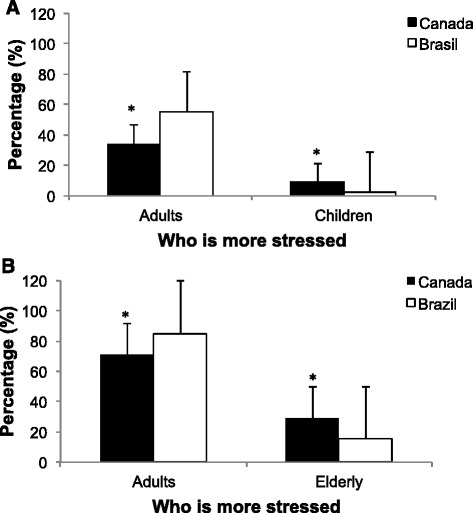
Percentages of Canadian and Brazilian individuals believe adults to be more susceptible to stress than **a** children and in the **b** elderly. * indicates p-values less than 0.01 between groups. The bars represent standard errors

To investigate the perceptions of daily stress experienced by children, adults, and the elderly, participants were asked who they felt were more susceptible to stress on a daily basis: adults or children; adults or the elderly. Here, it is important to note that many studies now show that stress is as deleterious for children and older adults as it is for adults [9]. The intra-group analysis showed that the majority of Canadian and Brazilian participants believed stress to be higher in adults compared to that encountered by children and older adults. However, the percentage of Canadian participants that reported children (*n* = 101; 20.8 %) and elderly (*n* = 142; 29.1 %) as groups of people who experience elevated levels of stress is significantly higher compared to Brazilians participants (children: *n* = 26; 4.0 %; elderly: *n* = 98; 15.1 %; *p* < 0.001) in the inter-group analysis. Figure 2.

Multivariate analysis showed that responses regarding who the participants felt were more susceptible to stress “Adults *vs.* Children” and “Adult *vs.* Elderly*”* were influenced by country group, age and gender (*p* < 0.001; Table 4).

**Table 4:** Multivariate regression results regarding “who is more stressed”

Model	Adult *vs* Children					Adult *vs* Elderly				
	β	*p*	R^2^	F	*p*	β	p	R^2^	F	*p*
*Model 1*			0.095	39.4	<0.001			0.072	28.8	<0.001
Country group	−**0.208**	<0.001				−**0.122**	<0.001			
Age	**0.142**	<0.001				**0.121**	<0.001			
Gender	**−0.064**	0.031				**−0.144**	<0.001			

Significant β values (*p* < 0.05) are in boldface

### Characteristics leading to stress

**Fig. 3:**
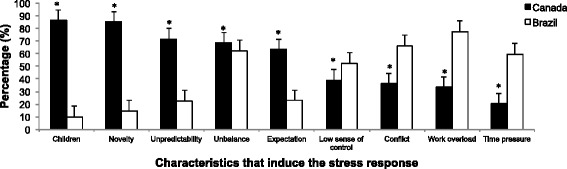
The percentage of Canada and Brazil participants identifying characteristics capable of inducing a stress response. * indicates p-values less than 0.027. The bars represent standard errors

Participants were asked to indicate which of the following elements elicit a stress response: time pressure; novelty; conflict; little control over situation; unpredictability; threat to ego, lack of balance between resources and demands; work overload; expectations from others; children. Here, it is important to remind the reader that the four characteristics that have been shown in the scientific literature to lead to a stress response are novelty, unpredictability, threat to ego and lack of sense of control [6, 7]. When comparing the groups, we found that the Canadian participants reported the following elements the most frequently: children (*n* = 428; 86.5 %), novelty (*n* = 422; 85.3 %), unpredictability (*n* = 354; 71.7 %), unbalance (*n* = 340; 68.7 %) and expectation from others (*n* = 315; 63.6 %). On the other hand, work overload (*n* = 506; 77.4 %), conflict (*n* = 434; 66.4 %), time pressure (*n* = 391; 59.8 %) and low sense of control (*n* = 343; 52.5 %) were predominantly indicated by the Brazil group. Interestingly, only the participants from Canada predominantly indicated the elements that represent the scientific psychological determinants of stress (novelty, unpredictability, low sense of control and social evaluation), as characteristics that induce a stress response. Figure 3.

Accordingly, multivariate analysis controlling for age and gender showed that country group predict the responses regarding all the elements capable of triggering a stress response (*p* < 0.001). Age combined with country group influenced “social-evaluative threat” and “conflict” assumptions regarding their potential to elicit a stress response. The older the individual the higher the number of participants who considered social-evaluative threat as stressful (β = −0.065; *p* = 0.031). For those younger, “conflict” is the psychological determinant of stress response (β = 0.110; *p* < 0.001; Table 5). Novelty, uncontrollability and unbalance were influenced by both country group and gender. While women perceived unbalance and uncontrollable events as stressful (β = 0.110; *p* < 0.001), men reported novelty as the psychological determinant of stress (β = 0.110; *p* < 0.001; Table 5).

**Table 5:** Multivariate regression results regarding scientific and public knowledge about determinants of stress response

Dependent variable	Independent Variable					
	Country group	Age	Gender			
	β (p)	β (p)	β (p)	R^2^	F	*p*
*Scientific determinants*						
Novelty	**0.702 (<0.001)**	−0.015 (0.542)	**0.044 (0.038)**	0.495	372.1	<0.001
Unpredictability	**0.497 (<0.001)**	−0.009 (0.167)	−0.016 (0.548)	0.216	372.4	<0.001
Uncontrollability	**−0.132 (<0.001)**	−0.023 (0.481)	**−0.061 (0.037)**	0.021	12.4	<0.001
Social-evaluative threat	**0.337 (<0.001)**	**−0.065 (0.031)**	0.013 (0.642)	0.134	88.3	<0.001
*Public knowledge of determinants*						
Time pressure	**−0.396 (<0.001)**	−0.033 (0.291)	−0.015 (0.610)	0.157	211.5	<0.001
Conflict	**−0.261 (<0.001)**	**0.110 (<0.001)**	0.047 (0.113)	0.102	64.8	<0.001
Unbalance	**0.063 (0.031)**	−0.02 (0.545)	**−0.129 (<0.001)**	0.02	11.9	<0.001
Work overload	**−0.446 (<0.001)**	0.03 (0.326)	0.013 (0.640)	0.199	281.9	<0.001
Children	**−0.770 (<0.001)**	−0.006 (0.783)	0.000 (0.990)	0.592	1654.7	<0.001

^a^ Not included in the model 2 due to non significant effect in the model 1. Significant β values (*p* < 0.05) are in boldface.

## Discussion

The results of the current study report some important cross country data on the general public’s conceptions about the notion of stress. Both the Canadian and Brazilian groups presented misconceptions of stress concepts and the potential factors capable of inducing a stress response, thus suggesting a lack of public understanding about the science of stress in both countries.

Contrary to the scientific definition of stress conceptualized as being an adaptive bodily response to physiological and psychological stressors, there were participants in both Canadian and Brazilian groups who perceived stress to be a harmful and negative experience, and who believed that a totally stress-free life is beneficial. Participants’ responses also showed misconceptions of stress as they were unable to correctly identify the scientific characteristics capable of eliciting a stress response. A number of participants from both groups believe that “time pressure” and “work overload” are major elements that induce a stress response. These findings support the presence of a lack of public understanding of the scientific notions of stress despite the development of extensive research in this field over the last decades. This results show that major efforts in knowledge transfer should be put into place in order to better inform the public about contemporary scientific results on the determinants and consequences of human stress.

Some historical ambiguities may partially explain why the general and scientific communities still hold different concepts about stress. Previous research on stress that occurred during the first and second world wars was characterized by several debates and concept controversies around the psychological and physiological concepts of stress [19]. Historically, these debates and controversies focused mainly on the determinants capable of triggering a stress response (critical life events *versus* hassles and uplifts, physiological *versus* psychological and sociocultural and so on) [20]. In fact, the term “*stress”* has been used in a somewhat ambiguous fashion over the years and by different scientific disciplines. Additionally, several stress studies have been supported by findings based on work-related stress (e.g., stressful professions) and illness models (stress-related disorders), which may have contributed to the misconception that stress represents a medical condition or mainly a work-related disorder (work overload and inability to manage resources and demands).

Another factor that could explain the lack of public understanding of stress may be associated with models of public communication of science. Important communication projects developed by government agencies or other communication industries to address scientific information for the general public use a top-down and passive approach where the public is exposed to simplified information on a given topic. This communication approach, known as the “deficit model” is a model in which it is postulated that a deficit of knowledge exists in the general society that must be filled by communication experts, without considering the context or the relevance of certain knowledge in exposed people [21, 22]. It has been shown that communication activities using this approach have not changed the percentage of the public “correctly” answering a series of factual questions suggesting that the “deficit” model may need additional models to transfer scientific knowledge toward the public [22]. In the neuroscience field, Illes et al. [23] reported three courses of action to facilitate dialogue with general public: *“a cultural shift that explicitly recognizes and rewards public outreach, the identification and development of neuroscience communication experts, and ongoing empirical research on the public communication of neuroscience*” [23].

Other different approach to communicate scientific data on stress to the general public is through the development of psychoeducational programs aimed at specific populations. New research has increasingly examined the effects of stress-management techniques in adults [24–28] and more recently in adolescents [29]. In the field of psychoeducational programs, three types of prevention programs have been developed to target different populations. Universal programs are usually presented to all individuals regardless of symptoms and are often designed to build resiliency and/or enhance general mental health [29]. Selective programs are presented to individuals who are at risk of developing a mental health problem as a function of particular risk factors related to stress, while indicated programs are delivered to individuals who present mild or severe symptoms of a mental health disorder [30].

Because universal interventions have the advantage of avoiding the stigma of singling out individuals for treatment [31, 32], some universal programs on stress have been developed for adolescents. For example, the *Gatehouse Project* was created to reduce stressors in the environment by creating a more inclusive classroom environment with a focus on improving interpersonal bonds [33, 34]. Similarly, the *Transition Club Project* was developed to help students gradually acclimate to the secondary school environment through pre-transition exposure [35]. In a more recent study, our group exposed 504 adolescents to the *DeStress for Success Program*, a psychoeducational program based on scientific data from human stress research. The results of the study showed that exposure to the program led to significant decreases in stress hormone levels and depressive symptoms, particularly in adolescents who started the school year with high levels of anger [36]. Interestingly, very recent evidence has shown that individuals instructed to perceived stress arousal as a beneficial body reaction instead of the automatically negative perception showed better cardiovascular, cognitive and social-interactive outcomes in a evaluative stress task. Specifically, those individuals displayed better cardiac efficiency, low cardiovascular resistence, less negative affect and threat-related attentional bias and performed the evaluative task more efficiently compared to controls. These findings suggesting that changing the way we view stress may improves affective experiences and promote more adaptive physiological and psychological response [15–17].

Altogether, these results show that psychoeducational program targeting specific populations who are active participants in the knowledge exchange process can have important psychological and physiological effects on helping individuals learn about stress, which in turns helps them manage the stress response and its potential deleterious effects on physical and mental health.

The results of the study also showed that even controlling for demographic characteristics Brazilian and Canadians’ science-based knowledge of the characteristics that induce a stress response were different. The percentage of “incorrect” answers about stress concepts was approximately three-fold higher in the participants from Brazil compared to Canada. Additionally, groups differed from each other regarding the elements capable of eliciting a stress response. In the Canadian group “children”, “novelty” and “unpredictability” were the major characteristics of a situation thought to elicit a stress response while the Brazilian participants indicated “time pressure”, “work overload” and “conflict” as the major stressors in daily life showing a pattern of elements almost reverse between Brazil and Canada. It has been extensively demonstrated that only psychological stressors: novelty, unpredictability, uncontrollability and social-evaluative threat, can activate the HPA axis triggering significant increase of cortisol levels above baseline values [6, 7].

These findings suggest that a country’s context and characteristics could modulate the stress beliefs in the general public and there are some explanations to support this interpretation.

Low levels of education can account for the difficulties in accessibility to scientific information as well as the misconceptions about stress [37, 38]. In Brazil, the high illiteracy rate (12.4 %), low education level (7.9 years) as well as inequality of access to education could partially explain the higher frequency of misconceptions about stress [39]. Moreover, socioeconomic resources, and environments may influence perceptions about threatening events as well as emotional and cognitive responses [40]. Brazilians experience long work weeks (39.4 h on average and 44 h at maximum), work overload (43.6 % of Brazilians have to do overtime), informant employment (10.7 % of Brazilians work without the coverage of labor laws and employment insurance) as well as low salaries associated with low purchasing [41]. In addition, Brazilians are routinely faced with social adversities such as low access to public health services, education and transportation as well as frequent exposure to social tension, violence, crowding and vehicular traffic. Finally, women in BRIC countries, which include Brazil [42, 43], do not expect their careers to be put on the side by children. The role of extended families in their daily life as well as the availability of low cost domestic help, both help to alleviate much of the pressure of balancing career and family [42–44]. On the other hand, in Canada where the focus is more on the nuclear family as an independent unit, individuals do not involve family as much. This could be a reason for the significant difference in the Canadian participants having reported “children” as being a situation that elicits a stress response when compared to the Brazilian participants [44].

Overall these environmental characteristics may explain the high percentage of Brazilian participants that reported “time pressure”, “work overload” and “conflict” as a source of stress. These results are in line with the country’s contextual influence on the public’s understanding of stress concepts.

Both Canadian and Brazilian participants exhibited the popular belief that young children and elderly are not stressed. Contrary to this belief, previous findings have shown that children and adolescents as well as older adults are capable as adults of experiencing stress [45–47]. A study has shown that young children can experience stress mainly as an influence of family environment on children’s secretion of stress hormones [47]. In fact, an increasing incidence of psychiatric problems during childhood as well cognitive deficits during older adulthood have been associated with stress showing how vulnerable these individuals are to the negative effects of stress [9, 48, 49].

Given the positive effects of psychoeducational stress programs on psychological and physiological measures of stress, it could be interesting to export some of these programs to the Brazilian populations.

Despite the interesting results that it provided, our study has some limitations. First, age differences were observed between groups and few male individuals were included in the study. Given that gender and age seems to easily transcend culture differences analysis of age and gender [43, 44] clusters may reveal additional findings. Regarding that, it should be highlighted that the country differences observed was associated stress knowledge even controlling for age and gender. Additional sociodemographic variables such as educational attainment, income inequality, and self-perception regarding socioeconomic status should be considered as moderate factors in future studies. Second, only Canadian and Brazilian participants were assessed which is a limitation to any generalization. Even though these countries represent North and South America societies, Brazil and Canada are relatively similar in terms of Western culture implying that a more broadly investigation across world, including Eastern countries, may reveal additional findings. Finally, given that cross-country differences may influence stress reactivity and diurnal rhythm of cortisol secretion [50], stress biomarkers under acute and chronic stressful conditions allied to perceived stress should be incorporated in future research aiming to contribute to further understanding of how cross-cultural differences may shape individuals perception and reaction to challenging and threatening contexts.

The results of this study are important because they show the existence of cross-country differences in the conception of stress. The historical conceptualization of stress as well as inadequate models of public communication of science may explain the current findings. In addition, given that the rate of misconceptions varied according to country, we also suggest that environmental characteristics may influence the public’s understanding of stress. In terms of methodological issues, our findings have provided important insights about the disparity between the public and scientific definitions of stress that could bias subjective stress assessment using questionnaires not regionally and/or culturally adapted. Finally, our findings may drive international policy marker discussions regarding wide word strategies to disseminate scientific stress knowledge in the lay public, specially during childhood and aging that represent critical periods in the window of vulnerability.

### Conclusions

Our results highlight the importance of publicizing the scientific knowledge about stress among the lay public, especially among children and elderly and those from underprivileged social context who are more vulnerable to the stress-related disorders.

## Abbreviations

BRICS, acronym that refers to the countries of Brazil, Russia, India, China and South Africa; HPA, hypothalamic-pituitary-adrenal; SAM, sympathetic-adrenal-medullary.

## Additional file

Additional file 1: Survey of Global Stress Knowledge. (PDF 77 kb)

## References

[1] National Science Board (2012). Science and Technology: Public Attitudes and Public Understanding, in Science & Engineering Indicators-2012.

[2] American Psychological Association. Stress in America 2010 report: key findings. Stress in AmericaTM Survey; 2010.

[3] Lupien SJ, Maheu F, Tu M, Fiocco A, Schramek TE (2007). The effects of stress and stress hormones on human cognition: implications for the field of brain and cognition. Brain Cogn.

[4] Seery MD (2011). Challenge or threat? Cardiovascular indexes of resilience and vulnerability to potential stress in humans. Neurosci Biobehav Rev.

[5] Lazarus RS (2006). Emotions and interpersonal relationships: toward a person-centered conceptualization of emotions and coping. J Person.

[6] Mason JW (1968). A review of psychoendocrine research on the sympathetic-adrenal medullary system. Psychosom Med.

[7] Dickerson SS, Kemeny ME (2004). Acute stressors and cortisol responses: a theorical integration and synthesis of laboratory research. Psychol Bull.

[8] Sapolsky RM, Romero LM, Munck AU (2000). How do glucocorticoids influence stress responses? Integrating permissive, suppressive, stimulatory, and preparative actions. Endocr Rev.

[9] Lupien SJ, McEwen BS, Gunnar MR, Heim C (2009). Effects of stress throughout the lifespan on the brain, behavior and cognition. Nat Rev Neurosci.

[10] McEwen BS (2007). Physiology and neurobiology of stress and adaptation: central role of the brain. Psysiol Rev.

[11] Statistics Canada. “How healthy are Canadians? 2001 annual report.” Health Reports, Special issue (Statistics Canada, catalogue no. 82-003-XIE) 12(3).

[12] Stewart WF, Ricci JA, Chee E, Hahn SR, Morganstein D (2003). Cost of lost productive work time among US workers with depression. JAMA.

[13] Ururahy G. O estresse custa caro às empresas. 2011. http://www.medriocheck-up.com.br/artigos//material/O%20estresse%20custa%20caro%20as%20empresas%20(2).pdf. Accessed 02 June 2016.

[14] World Health Organization. The World Health Report 2001 - Mental Health: New Understanding. http://www.who.int/whr/2001/en/whr01_en.pdf?ua=1. Accessed 02 June 2016.

[15] Beltzer ML, Nock MK, Peters BJ, Jamieson JP (2014). Rethinking butterflies: the affective, physiological, and performance effects of reappraising arousal during social evaluation. Emotion.

[16] Jamieson JP, Nock MK, Mendes WB (2013). Changing the conceptualization of stress in social anxiety disorder: affective and physiological consequences. Clin Psychol Sci.

[17] Jamieson JP, Nock MK, Mendes WB (2012). Mind over matter: reappraising arousal improves cardiovascular and cognitive responses to stress. J Exp Psychol Gen.

[18] World Health Organization (2012) World Health Statistics 2012: Global health indicators. http://apps.who.int/iris/bitstream/10665/44844/1/9789241564441_eng.pdf. Accessed 02 June 2016.

[19] Lupien SJ (2012). Well Stressed: Manage Stress Before it Turns Toxic.

[20] Monat A, Lazarus RS, Reevy G (2007). The Praeger Handbook on Stress and Coping.

[21] Ziman J (1991). Public understanding of science. Sci Tech Hum Values.

[22] Lewenstein BV (1992). The meaning of ‘public understanding of science’ in the United States after World War II. Public Understan Sci.

[23] Illes J, Moser MA, McCormick JB, Racine E, Blakeslee S, Caplan A (2010). Neurotalk: improving the communication of neuroscience research. Nat Rev Neurosci.

[24] Edwards D, Burnard P, Owen M, Hannigan B, Fothergill A, Coyle D (2003). A systematic review of the effectiveness of stress-management interventions for mental health professionals. J Psychiatr Ment Health Nurs.

[25] Gaab J, Blattler N, Menzi T, Pabst B, Stoyer S, Ehlert U (2003). Randomized controlled evaluation of the effects of cognitive-behavioral stress management on cortisol responses to acute stress in healthy subjects. Psychoneuroendocrinology.

[26] Gaab J, Sonderegger L, Scherrer S, Ehlert U (2006). Psychoneuroendocrine effects of cognitive-behavioral stress management in a naturalistic setting: a randomized controlled trial. Psychoneuroendocrinology.

[27] Murphy LR (1996). Stress management in work settings: a critical review of the health effects. Am J Heal Prom.

[28] Richardson KM, Rothstein HR (2008). Effects of occupational stress management intervention programs: a meta-analysis. J Occupation Health Psychol.

[29] Barrett P, Turner C (2001). Prevention of anxiety symptoms in primary school children: preliminary results from a universal school-based trial. Br J Clin Psychol.

[30] Donovan CL, Spence SH (2000). Prevention of childhood anxiety disorders. Clin Psychol Rev.

[31] Rapee RM, Wignall A, Sheffield J, Kowalenko N, Davis A, McLoone J (2006). Adolescents’ reactions to universal and indicated prevention programs for depression: perceived stigma and consumer satisfaction. Prev Sci.

[32] Sheffield JK, Spence SH, Rapee RM, Kowalenko N, Wignall A, Davis A (2006). Evaluation of universal, indicated, and combined cognitive-behavioral approaches to the prevention of depression among adolescents. J Consult Clin Psychol.

[33] Patton GC, Glover S, Bond L, Butler H, Godfrey C, Di Pietro G (2000). The Gatehouse Project: a systematic approach to mental health promotion in secondary schools. Aust N Z J Psychiatry.

[34] Patton GC, Bond L, Carlin JB, Thomas L, Butler H, Glover S (2006). Promoting social inclusion in schools: a group-randomized trial of effects on student health risk behavior and well-being. Am J Public Health.

[35] Humphrey N, Ainscow M, Transition Club (2006). Facilitating learning, participation and pyschological adjustment during the transition to secondary school. Eur J Psychol Educ.

[36] Lupien SJ, Ouellet-Morin I, Trepanier L, Juster RP, Marin MF, Francois N (2013). The DeStress for Success Program: effects of a stress education program on cortisol levels and depressive symptomatology in adolescents making the transition to high school. Neuroscience.

[37] Macintyre SS, Maciver S, Solomon A (1993). Area, class and health: should we focusing on places or people. J Soc Politic Psychol.

[38] Troutt DD (1993). The thin red line: how the poor still pay more.

[39] Síntese de indicadores sociais. Ministério do Planejamento, Orçamento e Gestão, Instituto Brasileiro de Geografia e Pesquisa (IBGE). 2002. http://www.ibge.gov.br/home/estatistica/populacao/condicaodevida/indicadoresminimos/indic_sociais2002.pdf. Accessed 02 June 2016.

[40] Coriell M, Adler NE. Social ordering and healthy. In: McEwen B, Goodman HM. Handbook of physiology: coping with the environment neural and endocrine mechanisms. New York: Oxford University Press; 2001.

[41] Instituto de Pesquisa Econômica Aplicada (IPEA). Carga horária de trabalho: evolução e principais mudanças no país. 2009. http://www.en.ipea.gov.br/agencia/images/stories/PDFs/comunicado/090729_comunicadoipea24.pdf. Accessed 02 June 2016.

[42] Elder M, Leahy J, Rumsey J, Anderlini J. Who’s who: Bric leaders take their place at the top table. 2008. Financial Times, London. http://www.ft.com/cms/s/0/d31392b2-89ca-11dd-8371-0000779fd18c.html#axzz3W4LbIPsr. Accessed 02 June 2016.

[43] Instituto Brasileiro de Geografia e Estatística (IBGE) (2011). Censo Demográfico 2010: características da população e dos domicílios - resultados do universo.

[44] Andersen ML, Taylor HF (2007). The extended family may live together for many reasons, help raise children, support for an ill relative, or help with financial problems.

[45] Lohman BJ, Jarvis PA (2000). Adolescent stressors, coping strategies, and psychological health studied in the family context. J Youth Adolesc.

[46] Lupien SJ, Lecours AR, Schwartz G, Sharma S, Meaney MJ, Nair NPV (1996). Longitudinal study of basal cortisol levels in healthy elderly subjects: evidence for subgroups. Neurobiol Aging.

[47] Lupien SJ, King S, Meaney MJ, McEwen BS (2001). Can poverty get under your skin? Basal cortisol levels and cognitive function in children from low and high socioeconomic status. Dev Psychopathol.

[48] Hudziak JJ, Rudiger LP, Neale MC, Heath AC, Todd RD (2000). A twin study of inattentive, aggressive, and anxious/depressed behaviors. J Am Acad Child Adolesc Psychiatry.

[49] Angold A, Erkanli A, Silberg J, Eaves L, Costello EJ (2002). Depression scale scores in 8-17-year-olds: effects of age and gender. J Child Psychol Psychiatry.

[50] Souza-Talarico JN, Plusquellec P, Lupien SJ, Fiocco A, Suchecki D (2014). Cross-country differences in basal and stress-induced cortisol secretion in older adults. Plos One.

