# Supplementary material for: Cross-country discrepancies on public understanding of stress concepts: evidence for stress-management psychoeducational programs
Source: BMC Psychiatry. 2016 Jun 3;16:181. doi: 10.1186/s12888-016-0886-6 (PMC4893292; doi:10.1186/s12888-016-0886-6)
Supplement: Additional file 1: — Survey of Global Stress Knowledge. (PDF 77 kb) [file 12888_2016_886_MOESM1_ESM.pdf]

Age: _______
Female: ____ Male: ____

Survey of Global Stress Knowledge:

The Centre for Studies on Human Stress and its division concerned with Observations of Stress in Quebec (OSQ) are currently interested in discerning the general conceptions of stress held by Quebecers. In order to decipher your global opinions about stress, we would greatly appreciate obtaining your responses to the following questions outlined below. These responses will remain completely confidential and because we do not require your name it will not be possible to distinguish your responses from those comprising the overall survey.

Thank you for your help!

1. Stress is bad.

   TRUE FALSE

2. According to you, out of adults and children, who is more susceptible to stress on a daily basis? Please circle your answer.

   ADULTS CHILDREN

3. According to you, out of adults and elderly people, who is more susceptible to stress on a daily basis? Please circle your answer.

   ADULTS ELDERLY

4. According to you, what are the 4 most important characteristic(s) that induce stress? Please circle your answer(s).
   (a) Time pressure
   (b) Novelty
   (c) Conflict
   (d) Little control over situation
   (e) Unpredictability
   (f) Lack of balance between resources and demands
   (g) Work overload
   (h) Expectations from others
   (i) Children

5. Men are more likely than women to "Burn-Out" due to stress.

   TRUE FALSE

6. Being totally stress-free is a good thing.

   TRUE FALSE

7. Stress can cause:

(a)	Alzheimer's Disease	True ____	False ____
(b)	Obesity	True ____	False ____
(a)	Schizophrenia	True ____	False ____
(b)	Physical Pain	True ____	False ____

Age: _______

Female: ____ Male: ____

(d)	Depression	True	____	False	____
(e)	Problems with Digestion	True	____	False	____
(f)	Alcoholism and Drug Abuse	True	____	False	____
(g)	Attentional Problems	True	____	False	____
(h)	The Flu	True	____	False	____
(i)	Mental Retardation	True	____	False	____
(j)	Insomnia	True	____	False	____

8. Women are more likely than men to develop depression due to stress.

TRUE FALSE

9. Can you normally recognize if you are feeling stressed?

YES NO

10. If you answered YES to the above question, how do you know when you are stressed? Please circle the 4 most important choices.
(a) Feeling tired
(b) Unable to fall asleep
(c) Getting sick
(d) Becoming irritable
(e) Unable to make rational decisions
(f) Feeling your own heart beating
(g) Becoming conscious of your own breathing
(h) Lacking motivation or drive
(i) Feeling anxious
(j) Becoming more impatient
(k) Others (Please Specify)____

11. Can you normally recognize when people around you (e.g. family members, friends, co-workers) are stressed?

YES NO

12. If you answered YES to the above question, how do you know when people around you are stressed? Please circle the 4 most important indicators.
(a) They appear tired and fatigued
(b) They become ill
(c) They are irritable
(d) They make irrational decisions
(e) They lack motivation or drive
(f) They appear sad

13. On a scale from 0 to 10, how stressed (on average) would you rate yourself in comparison to others around you? 0 = no stress at all; 10 = highly stressed. Please circle the number.

0 1 2 3 4 5 6 7 8 9 10

Age: _______
Female: ____ Male: ____

14. On a scale from 0 to 10, how stressed would you say the general Quebec population is? 0 = no stress at all; 10 = highly stressed. Please circle the number.

0 1 2 3 4 5 6 7 8 9 10

15. On a scale from 0 to 10, how well do you think you can cope with stress compared to people around you? 0 = unable to cope with any kind of stress at all; 10 = able to cope with any kind of stress.

0 1 2 3 4 5 6 7 8 9 10

16. On a scale from 0 to 10, how well do you think people in Quebec cope with stress?
0 = unable to cope with any kind of stress at all; 10 = able to cope with any kind of stress.

0 1 2 3 4 5 6 7 8 9 10

17. Please rate from 1 (causes very little stress) to 10 (causes me a lot of stress), the extent to which the following items can cause or exacerbate your level of stress.

(a) Expectations of others ____
(b) Expectations of yourself ____
(c) Work-related responsibilities ____
(d) Household responsibilities ____
(e) Trying to change others ____
(f) Sad memories ____
(g) Negative memories ____
(h) When others blame me for various things ____
(i) Problems anticipating things ____
(j) Dealing with the unpredictable ____
(k) Dealing with novelty ____
(l) Driving in traffic
(m) Work-related meetings ____

Thank you!

Sonia Lupien Ph.D.
Director, Centre for Studies on Human Stress
